# Analysis and Design of Single-Ended Resonant Converter for Wireless Power Transfer Systems

**DOI:** 10.3390/s22155617

**Published:** 2022-07-27

**Authors:** Qiqi Li, Shanxu Duan, Han Fu

**Affiliations:** State Key Laboratory of Advanced Electromagnetic Engineering and Technology, Huazhong University of Science and Technology, Wuhan 430074, China; lqq@hust.edu.cn (Q.L.); duanshanxu@hust.edu.cn (S.D.)

**Keywords:** analytical model, single-ended resonant converter, wireless power transfer, zero-voltage switching

## Abstract

Single-ended resonant converters such as Class-E inverters have been widely considered as a potential topology for small- and medium-power wireless power transfer (WPT) applications, which feature compact circuits, low switching losses, and cost benefits, as they only use a low-side switch with a simple gate driver. However, there remains a practical challenge in the design of voltage stress, efficiency, and power density. In this paper, a single-ended resonant converter with a primary parallel resonant-matching network is investigated to absorb the bulky input-choke inductors of the Class-E inverters into the coil inductance. The analytical expressions for all the converter parameters are derived based on time-domain resonant waveforms, including: (1) analysis of critical zero-voltage switching (ZVS) conditions and (2) power transfer capabilities under the given maximum switch voltage stress. Furthermore, this paper elaborates on the design methodology of the proposed single-ended resonant converters, and an optimal operating point is chosen to ensure soft-switching operation and rated power. Finally, the accuracy of the proposed model is verified by simulation and experimental results.

## 1. Introduction

Wireless power transfer (WPT) brings about a convenient and safer means of supplying power to electronic devices. It is not only drawing increasing attention from academia but has been popularized in daily life, such as in portable products [1], medical implants [2], and electric vehicles [3,4,5,6]. In general, using a high switching frequency can reduce the size of passive components and improve power density for WPT systems. However, the resultant switching losses and parasitic oscillations will impose adverse effects on the operation and performance of the converter, especially for those containing inefficient loosely coupled transformers. For this reason, applying the soft-switching technique has become an increasing trend in WPT systems. The typical soft-switching inverter configuration is composed of a high-frequency inverter and a resonant tank. The AC voltage generated by the inverter excites the compensators in the matching network and the resonance characteristics decide the zero-voltage switching (ZVS) conditions of the switching devices. It is very important to choose a suitable soft-switching topology for WPT systems, as this will make it especially appropriate for cost-sensitive applications such as household electric appliances, automatic logistics robots, and portable equipment.

The full-bridge (FB) voltage-source inverter (VSI) is regarded as the desired solution for high-power WPT applications such as electric vehicle chargers due to its powerful transfer capability, low voltage stresses, and high controllability. Various types of matching networks have been proposed and analyzed for FB VSIs. Because of the current source characteristic of inductors, series compensators could be directly connected to VSIs, which fosters some basic compensation topologies such as the series–series [7], series–parallel [8], and series–none [9]. Extra inductors and capacitors were also introduced to form inductor–capacitor–inductor [10], inductor–capacitor–capacitor [11], and hybrid compensation networks [12,13] to further improve efficiency and reduce reactive power. In [14], a half-bridge Class-D inverter was used for kilowatts power-level WPT applications, with an optimized design method presented to attenuate the influence of the parasitic elements. A push–pull parallel resonant converter-based bi-directional WPT system was also proposed to provide a more efficient solution [15].

Although widely recognized in high-power scenarios, the bridge topology is less suitable for small- and medium-power WPT systems. It has at least two switches, which will not only require more sophisticated control but also raise production costs. By contrast, the single-ended topology where the converter contains a single low-side switch is known to exhibit good performances from kilohertz (kHz) to megahertz (MHz). It shows not only a low turn-on switching loss but also a low turn-off switching loss, since the resonant capacitor delays the voltage increase of the switch [16].

The Class-E inverter is one of the most common single-ended resonant converters. Many optimized design and control methodologies have been proposed to reduce switching losses and improve efficiency [17,18,19]. However, there remains a practical challenge in the balance of voltage stress, output power, and efficiency, motivating research on optimization methodologies. In [20], a compound voltage-clamped Class-E inverter with an auxiliary was proposed to further improve the performance and decrease the volume of the passive components. Unfortunately, the auxiliary needs a high-side driver, which aggravates its complexity. In [21], a push–pull structure and coupled-choke inductors were introduced into the Class-E inverter to increase output power. In [22], a hybrid inverter named Class-EF was proposed. This inverter improves switch voltage and current waveforms of Class-F inverters with the efficient switching of Class-E inverters.

**Figure 1:**
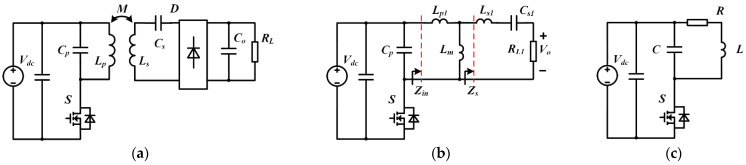
Typical configuration of the single-ended WPT systems: (**a**) single-ended resonant converter; (**b**) the transformer version equivalent circuit; (**c**) simplified model.

The input-choke inductors are needed in the above single-ended resonant converters, which is one of the factors that limit the converters’ performance. To further reduce the number of passive components, the high-frequency single-ended resonant converter, inspired by the induction-heated cooking appliances in [23], can be typically configured as in Figure 1a. Compared to the Class-E topologies, this topology replaces the input-choke inductor with a coil inductance *L_p_*, and a primary compensation capacitor *C_p_* is added to achieve its ZVS and delays the increase in the switch voltage. However, there are few discussions on the analysis of this converter and its design methodology. Therefore, this paper is devoted to presenting a detailed discussion of the current topology, with the analytical derivation of the ZVS condition given to guide the parameter design of WPT systems.

This paper aims to analyze the behavior of the single-ended resonant converter with parallel-series (PS) compensation for wireless charging applications. The analysis of the equivalent circuit of the converter is illustrated in Section 2. The characterization of the primary parallel resonance is analytically derived in Section 3, including resonance period calculation, critical ZVS conditions, and the voltage stress of key components. The detailed procedures to design an optimal parameter are developed based on the above analysis in Section 4. Then, Section 5; presents the simulation and experimental results to validate the analytical model. Finally, Section 6; draws the conclusions.

## 2. Modeling of a Single-End Resonant Converter

Figure 1a shows the typical topology of the single-ended resonant converter. In the circuit, *V_dc_* is the DC supply voltage source, *S* is the power switch, *C_o_* is the output filter capacitor, and *R_L_* is the load resistance. The compensation network is composed of a parallel capacitor *C_p_*, a primary self-inductor *L_p_*, a secondary self-inductor *L_s_*, and a series capacitor *C_s_*. An FB-rectifier circuit is placed between the compensation network and the output filter. Figure 1b shows the equivalent circuit, where the mutual inductance is replaced by the T-type decoupled-transformer model, and the secondary impedance is transferred to the primary side of the transformer. The parameters of the transformer consist of the coupling coefficient *k*, turn ratio *n*, magnetizing inductance *L_m_*, and the primary and secondary leakage inductances *L_p1_* and *L_s1_*.

The inductance can be expressed as Equations (1) and (2).

(1) $$L_{m}=kL_{p}$$

(2) $$L_{p1}=L_{s1}=(1-k)L_{p}$$

In Equation (1), the coupling coefficient $k=M/\sqrt{L_{p}L_{s}}$. The equivalent secondary resonant capacitance *C_s_*_1_ and the AC load *R_L_*_1_ are given as Equations (3) and (4).

(3) $$C_{s1}=n^{2}C_{s}$$

(4) $$R_{L1}=\frac{8n^{2}R_{L}}{\pi^{2}}$$

Parameter *n* is the transformer turn ratio, the value satisfies Equation (5).

(5) $$n=\sqrt{L_{p}/L_{s}}$$

The impedance of the secondary side at any operating condition is expressed in general as Equation (6).

(6) Zs=Re(Zs)+jIm(Zs)=RL1+jωs( Ls1+Cs1)

In Equation (6), $\omega_{s}=2\pi f_{s}$, and *f_s_* is the switching frequency of the converter. The input impedance *Z_in_* of the loosely coupled transformer with a complex load impedance is shown as Equation (7).

(7) $$Z_{in}=j\omega_{s}\;L_{p1}+\frac{j\omega_{s}\;L_{m}\;Z_{s}}{Z_{s}+j\omega_{s}\;L_{m}}$$

Substituting (6) into (7), the real part of Equation (7) is the transformer input resistance, and the imaginary part of Equation (7) is the transformer input reactance. The results are shown in Equations (8) and (9).

(8) Re(Zin)=ωs2 Lm2 RL1RL12+a02

(9) Im(Zin)=ωs Lp1+ω sLm RL12+a0ωs Lm(a0−Lm)RL12+a02

In (8) and (9), *a*_0_ is a simplified expression shown as Equation (10).

(10) $$a_{0}=\omega_{s}\;L_{s1}-\frac{1}{\omega {\;}_{s}C_{s1}}+\omega_{s}\;L_{m}$$

Figure 1c shows the simplified circuit model of the single-ended WPT system, with the equivalent circuit parameters computed as follows (Equations (11) to (13)):

(11) $$C=C_{p}$$

(12) R=Re(Zin)

(13) L=Im(Zin)ωs=Lp1+Lm RL12+a0Lm(a0−Lm)RL12+a02

The simplified model is used in the subsequent analysis. It is assumed that the output capacitance, ON-resistance and body-diode forward voltage of the MOSFET can be ignored. It should be noted that the parallel compensation must be connected to the primary switch to achieve ZVS, but the secondary compensation network could be selected by load characteristics. Although only the PS compensation has been analyzed in detail in this paper, the proposed method is also applicable to the parallel–parallel (PP) compensation topology.

**Figure 2:**
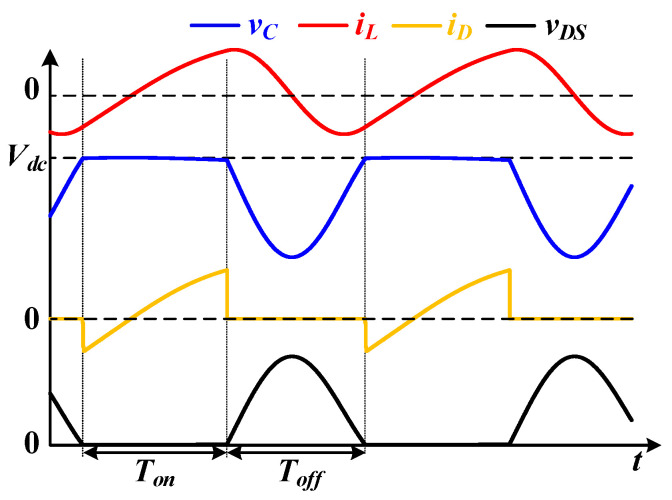
Idealized current and voltage waveforms.

The idealized current and voltage waveforms of the converter are shown in Figure 2, which can explain the operation principle of the converter. Here, *i_L_* is the current through the inductor, *v_C_* is the voltage on the parallel capacitor, *v_DS_* is the drain-to-source voltage of the MOSFET, and *i_D_* is the current through the MOSFET. From Figure 2, it can be discovered that the MOSFET switches when the parallel capacitor voltage is equal to the input voltage, which contributes to its low switching-loss feature. By selecting the capacitor voltage *v_C_* and the inductor current *i_L_* as state variables, the state equations for the *T_off_* period can be expressed as Equation (14).

(14) $$\left\{ \begin{array}{l} LC\frac{d^{2}v_{C}}{d^{2}t}+RC\frac{dv_{C}}{dt}+v_{C}=0\\ LC\frac{d^{2}i_{L}}{d^{2}t}+RC\frac{di_{L}}{dt}+i_{L}=0 \end{array}\right.$$

The characteristic equation can be derived as Equation (15).

(15) $$s=\beta \pm j\omega =-\frac{R}{2L}\pm j\sqrt{\frac{1}{LC}-\frac{R^{2}}{4L^{2}}}$$

As a result, solutions of the differential equations can be computed as Equation (16), with the coefficients *a*_1_–*a*_4_ obtained from different initial states.

(16) $$\{\begin{array}{c} v_{C}=e^{\beta t}(a_{1}\cos\omega t+a_{2}\sin\omega t)\\ i_{L}=e^{\beta t}(a_{3}\cos\omega t+a_{4}\sin\omega t) \end{array}$$

## 3. Characteristic Analysis

The equivalent model of the single-ended resonant converter is given in Section 2. To optimize the parameter design of the system, the analytical derivation of the circuit is required.

### 3.1. ZVS Analysis

**Figure 3:**
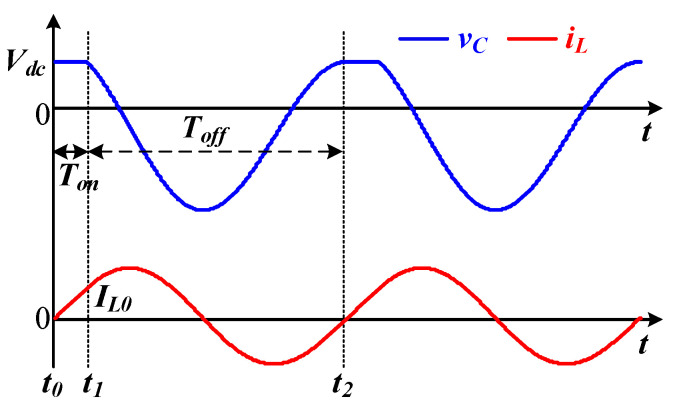
Voltage and current waveforms in critical ZVS conditions.

The critical ZVS waveform is shown in Figure 3. The MOSFET remains turned-on during *t*_0_ to *t*_1_, with the inductor current rising to *I_L_*_0_. When the MOSFET turns off at the end of *t*_1_, the inductor resonates with the parallel capacitors during the *T_off_* period. If the voltage of the parallel capacitor *v_C_* and the inductor current *i_L_,* respectively, are equal to *V_dc_* and zero at *t*_2_, the ZVS can be precisely achieved. To facilitate the derivation of the coefficients *a*_1_-*a*_4_, *t*_2_ can be assumed to be zero. 

Thus, the initial conditions of the state variables can be expressed as Equation (17).

(17) $$\{\begin{array}{l} v_{C}\left(0\right)=V_{dc}\\ i_{L}\left(0\right)=0\\ \frac{dv_{C}}{dt}\left(0\right)=-\frac{i_{L}\left(0\right)}{C}=0\\ \frac{di_{L}}{dt}\left(0\right)=\frac{v_{C}\left(0\right)-i_{L}\left(0\right)R}{L}=\frac{V_{dc}}{L} \end{array}$$

By substituting (18) into (17), the coefficients *a*_1_–*a*_4_ can be solved as Equation (18).

(18) $$\{\begin{array}{l} a_{1}=V_{dc}\\ a_{2}=-\frac{\beta }{\omega }V_{dc}\\ a_{3}=0\\ a_{4}=(\omega +\frac{\beta^{2}}{\omega })V_{dc}C \end{array}$$

According to Figure 3, the critical switch-off duration *T_off_* for ZVS can be obtained by letting *v_C_*(−*T_off_*) = *V_dc_* and *i_L_*(−*T_off_*) = *I_L_*_0_. In other words, Equation (19) can be established.

(19) $$e^{-\beta T_{off}}(\cos\omega T_{off}+\frac{\beta }{\omega }\sin\omega T_{off})=1$$

Since (19) is a transcendental equation with respect to the unknown variable *T_off_*, it can be solved by a numerical method. Then, the initial inductor current *I_L_*_0_ can be calculated from (16) as Equation (20).

(20) $$I_{L0}=(\omega +\frac{\beta^{2}}{\omega })V_{dc}Ce^{-\beta T_{off}}\sin(-\omega T_{off})$$

By letting *i_L_*(*T_on_*) = *I_L_*_0_, the minimum switch-on duration *T_on_* can be expressed as Equation (21).

(21) $$I_{L0}=\frac{V_{dc}}{R}(1-e^{-T_{on}R/L})$$

By combining (16)–(21), the average input current *i_inavg_* under the critical ZVS condition can be acquired as Equation (22).

(22) $$\begin{array}{c} i_{inavg}=\frac{1}{T_{on}+T_{off}}\int_{0}^{T_{on}}i_{L}dt\\ \;=\frac{V_{dc}}{R(T_{on}+T_{off})}(T_{on}+\frac{L}{R}(e^{-T_{on}R/L}-1)) \end{array}$$

The input power in this condition is Equation (23).

(23) $$P_{in}=V_{dc}i_{inavg}$$

On the other hand, it can be seen from (21) that the inductor current *i_L_* rises from zero to *I_L_*_0_ during *T_on_*, and ZVS conditions can never be achieved if *I_L0_* is equal to $V_{dc}/R$. Therefore, to achieve ZVS, Equation (24) should be satisfied.

(24) $$\left\{ \begin{array}{l} e^{-\beta T_{off}}(\cos\omega T_{off}+\frac{\beta }{\omega }\sin\omega T_{off})=1\\ e^{-\beta T_{off}}(\omega +\frac{\beta^{2}}{\omega })\sin(-\omega T_{off})=1/RC \end{array}\right.$$

Combining (15) and (24), the equations can be simplified as Equation (25).

(25) $$\left\{ \begin{array}{l} \omega T_{off}=Q\ln(\frac{1}{4}+{\left(Q\right)}^{2})\\ \tan(\omega T_{off})=-2Q \end{array}\right.$$

*Q* is the quality factor of the RLC resonant network, which is defined as Q=ωL/R. The numerical solution of the equations can be solved as Equation (26).

(26) $$\left\{ \begin{array}{l} \omega T_{off}=4.9006\\ Q=\frac{\omega L}{R}=2.5579 \end{array}\right.$$

By combining (15) and (26), the converter can achieve ZVS only when the *R*, *L* and *C* parameters meet, as in Equation (27).

(27) $$\frac{\sqrt{L/C}}{R}>2.6063$$

It can be concluded from (27) that when the inductance *L* is too small, or the capacitance *C* and resistance *R* are too large, ZVS may not be achieved. Thus, (27) can be used as an auxiliary criterion for the ZVS analysis.

### 3.2. The Derivation of Maximum Drain-to-Source Voltage

Since the time-domain expression of *v_C_* has been derived as (16), by solving the minimum capacitor voltage *V_Cmin_*, the maximum input power can be obtained. On the other hand, the maximum power is limited by the voltage stress of the switch *V_DSmax_* due to Equation (28), and *V_Cmin_* < 0.

(28) $$V_{DS\max}=V_{dc}-V_{C\min}$$

**Figure 4:**
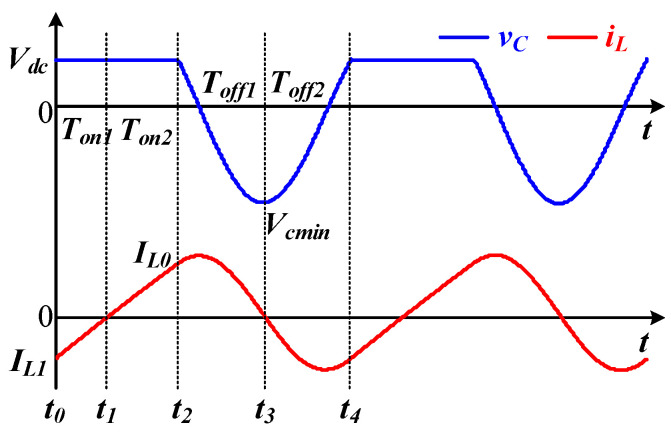
Voltage and current waveforms at maximum input power.

Figure 4 shows the typical operation waveforms at the maximum input power. 

When the inductor current *i_L_* is zero, the capacitor voltage *v_C_* is minimized, and the initial values of the state equations are given as Equation (29).

(29) $$\{\begin{array}{l} v_{C}\left(0\right)=V_{C\min}\\ i_{L}\left(0\right)=0\\ \frac{dv_{C}}{dt}\left(0\right)=-\frac{i_{L}\left(0\right)}{C}=0\\ \frac{di_{L}}{dt}\left(0\right)=\frac{v_{C}\left(0\right)-i_{L}\left(0\right)R}{L}=\frac{V_{C\min}}{L} \end{array}$$

By combining (16) and (29), the off-times *T_off1_* and *T_off2_* can be solved by letting *v_C_*(−*T_off_*_1_) = *v_C_* (*T_off_*_2_) = *V_dc_* as Equation (30).

(30) $$\left\{ \begin{array}{l} V_{C\min}e^{-\beta T_{off1}}(\cos\omega T_{off1}+\frac{\beta }{\omega }\sin\omega T_{off1})=V_{dc}\\ V_{C\min}e^{\beta T_{off2}}(\cos\omega T_{off2}-\frac{\beta }{\omega }\sin\omega T_{off2})=V_{dc} \end{array}\right.$$

The corresponding current *I_L0_* and *I_L1_* at *t*_1_ and *t*_2_ can be expressed as Equation (31).

(31) $$\left\{ \begin{array}{l} I_{L0}=(\omega +\frac{\beta^{2}}{\omega })V_{C\min}Ce^{-\beta T_{off1}}\sin(-\omega T_{off1})\\ I_{L1}=(\omega +\frac{\beta^{2}}{\omega })V_{C\min}Ce^{\beta T_{off2}}\sin\omega T_{off2} \end{array}\right.$$

Reselecting the origin at *t*_1_, the on-time can be obtained as Equation (32).

(32) $$\left\{ \begin{array}{l} T_{on1}=\frac{L}{R}\ln(1-I_{L1}R/(V_{dc}+V_{D}))\\ T_{on2}=-\frac{L}{R}\ln(1-I_{L0}R/V_{dc}) \end{array}\right.$$

By ignoring the voltage drop of the switch, the average input current is obtained and simplified as Equation (33).

(33) $$\begin{array}{l} i_{inavg}=\frac{1}{T_{on}+T_{off}}(\int_{-T_{on1}}^{0}i_{L}dt+\int_{0}^{T_{on2}}i_{L}dt)\\ =\frac{V_{dc}T_{on}}{R(T_{on}+T_{off})}+\frac{V_{dc}L}{R^{2}(T_{on}+T_{off})}(e^{-T_{on2}R/L}-e^{T_{on1}R/L}) \end{array}$$

In (33), *T_on_* = *T_on_*_1_ + *T_on_*_2_ and *T_off_* = *T_off_*_1_ + *T_off_*_2_. As a result, the input power under the *V_DSmax_* can be obtained by using (23).

## 4. Design Procedure

Based on the previous analysis, the detailed design procedure of the single-ended resonant converter can be summarized. To simplify the complexity of calculation, the key parameters of the equivalent circuit should be first designed. The principle of optimal design considered in this paper is to increase the ZVS range while maintaining the power capability. There are two specific constraints in the design process, i.e., ensuring the ZVS can be achieved at a light load *P_oL_*, and reaching the maximum input power *P_max_* at a given *V_DSmax_*. As explained in previous subsections.

**Figure 5:**
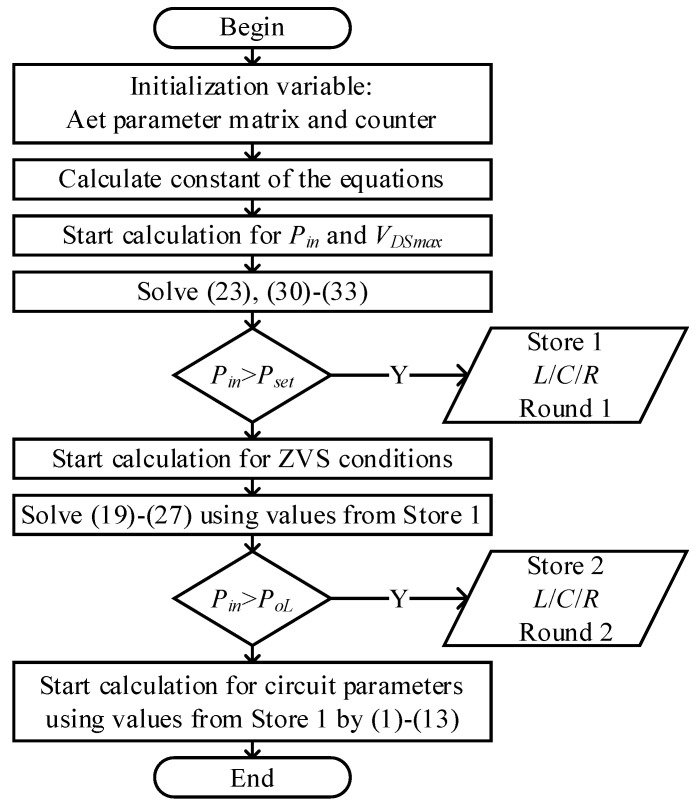
Flowchart of parameter design.

Figure 5 shows the flowchart of a parameter design process in MATLAB. If the range of parameters is given by system specification, the design procedure of the single-ended resonant converter can be developed as follows:

1. If the maximum drain-to-source voltage *V_DSmax_* is chosen, the simplified circuit parameters should comply with the relationships in (30)–(33) to make *P_max_* reachable. Calculate the detailed *L*, *R* and *C* values based on the *V_DSmax_* and the numeric range of *Q* to fulfill the target power and voltage stress;
2. Determine the required minimum *T_on_* and *T_off_* for critical ZVS conditions, and check whether the minimum input power meets the system requirements, (19)–(27);
3. Calculate *C_p_*, *L_p_*, *C_s_* and *L_s_* according to the obtained equivalent parameters by (1)–(13), with *L*, *R*, *C* and *Q* given.

**Figure 6:**
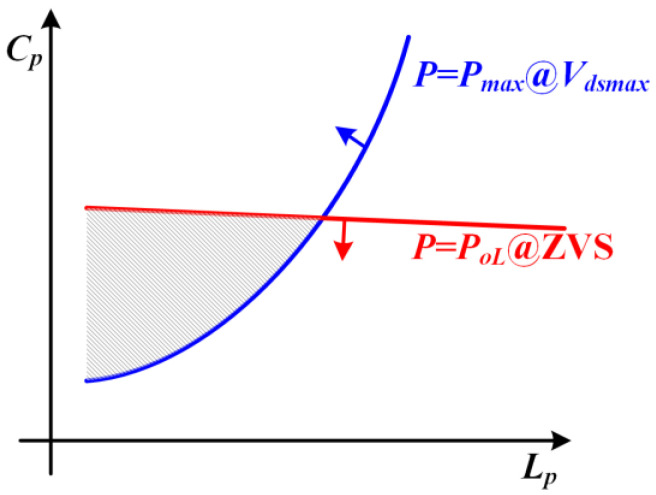
Input power *P_in_* as a function of primary self-inductance *L_p_* and parallel-resonant capacitance *C_p_,* illustrating the boundaries of ZVS and maximum drain-to-source voltage *V_DSmax_*.

According to the maximum power requirements and light-load ZVS conditions, the region of effective solutions for primary inductance and capacitance is shown in Figure 6.

The blue line indicates the *L_p_* and *C_p_* parameter combination when the load power reaches *P_max_* and the drain-to-source voltage is fixed to *V_DSmax_*. The red line indicates the *L_p_* and *C_p_* parameter combination when the load power reaches *P_oL_* with critical ZVS conditions satisfied. As seen in the figure, the shaded area is the operating region satisfying both the maximum power requirement and the constraint of light load ZVS. Within this range, the larger the *L_p_*, the smaller the primary current, which is conducive to reducing the current stress of the power semiconductor and the compensation capacitor, and beneficial to reduce the loss of resistance and improve conversion efficiency. Therefore, the optimized operation point can be selected at the right vertex of the region.

## 5. Simulation and Experimental Verification

In this section, the circuit shown in Figure 1 was designed, simulated, and tested to verify the proposed theory for single-ended resonant converters. The specifications of the single-ended resonant converter, design values, and the components selected for the simulations and experiments are provided in Table 1.

**Figure 7:**
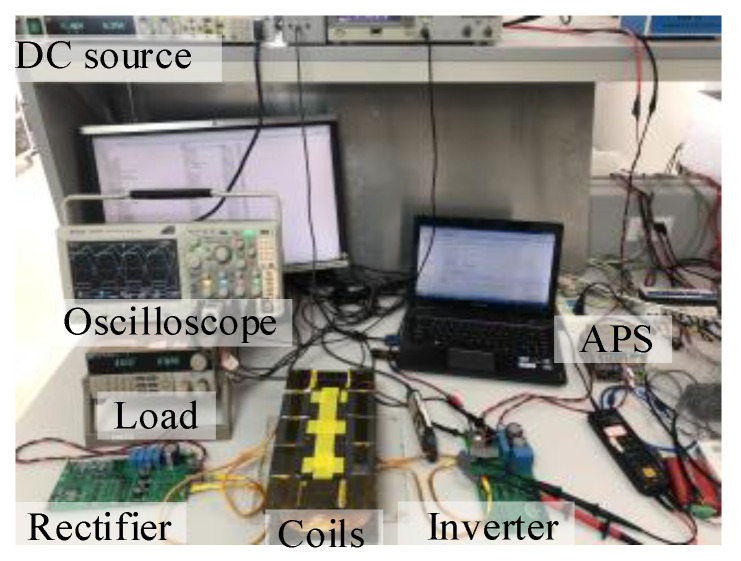
Photograph of the 100 kHz single-ended WPT systems.

The simulations were performed using Simulink. Figure 7 shows a photograph of the 100 kHz power prototype used for measurements. The self-inductance of the coils and their resistance were measured at 100 kHz and 2 V. For the experimental set-up, the distance between the two coils was fixed at 52 mm, and the turns of coil were adjusted to achieve the desired self-inductance. Note that the parameters of the experimental set-up were calculated based on an input power of 25 W and an actual output power of about 20 W with a cascade voltage-regulation module.

### 5.1. Steady-State Operation

**Figure 8:**
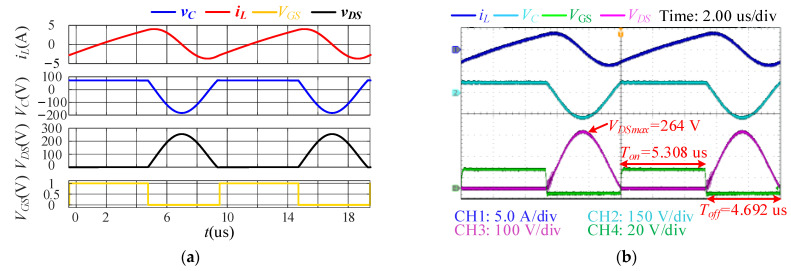
Simulation and experimentally obtained waveforms of single-ended resonant converter at *P_o_* = 25.495 W: (**a**) Simulated result; (**b**) experimental Result.

As mentioned, the single-ended resonant converter features not only a low turn-on switching loss but also a low turn-off switching loss because the resonant capacitor clamps the drain-to-source voltage. Figure 8 shows the gate-to-source, drain-to-source, coil current, and parallel capacitor voltage waveforms of the single-ended resonant converter obtained through simulations and experiments. The measured output power delivered to the load resistance *R_L_* is *P_o_* = 25.495 W. The measured average input power is *P_in_* = 27.3 W, while the design value is 25 W. Hence, the overall efficiency under the optimum operating conditions is *η* ≈ 93.39%.

The values of the designed parameters result in an optimal operation, where ZVS is achieved during both turn-on and turn-off. The designed maximum-voltage stress of the switch is 3.5*V_dc_* = 252 V, which is smaller than the measured value *V_DSmax_* = 264 V at the maximum-output power due to the ignorance of parasitic parameters. Moreover, it can be discovered that the switch voltage is negative and lower than the on-state voltage for a very short period of time. This negative-voltage freewheeling is caused by the body diode of the MOSFET, which increases the unnecessary conduction losses. The system efficiency can be improved by minimizing the turn-on delay of the switch after the negative-voltage freewheeling is detected. 

### 5.2. Critical ZVS Conditions

For the proposed single-ended resonant converter, various considerations need to be taken in the selection of switches, especially when operating at a small duty cycle. This is because the input capacitance, output capacitance, rise time, and fall time will affect the effective conduction time and thus the ZVS conditions. To minimize the influence of the nonlinear parasitic capacitance of switches, the SiC MOSFET SCT3080AL and Schottky barrier diode STPS30L60CT are applied for the converter, as they have relatively low parasitic capacitance and switching time at the rated voltage.

**Figure 9:**
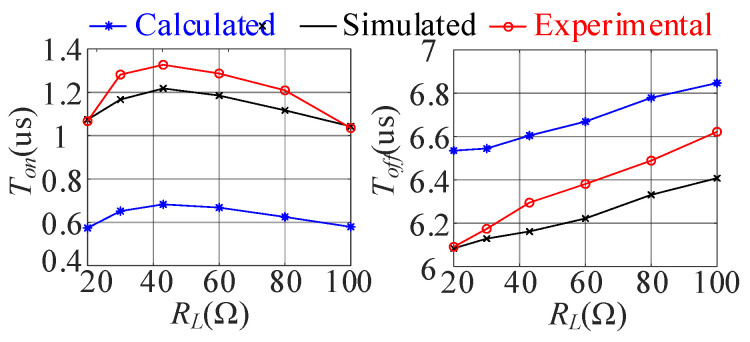
Comparison of calculated, simulated, and experimental critical ZVS conditions under different load resistances.

Figure 9 shows the calculated, simulated, and experimental turn-on times and turn-off times to achieve ZVS under different load resistances. The calculated values are obviously smaller than the simulation and experimental results because of ignoring the non-ideal factors, such as circuit resistance, parasitic capacitance, and the forward voltage drop of the body diode. From the analysis of Figure 9, it can be seen that the analysis result is consistent with the change trend of the measured values. It is desirable to leave enough margin in the design specification to take into account parasitic parameters and duty-cycle losses caused by the delayed time. For example, the minimum light-load power for ZVS can be slightly reduced to ensure a robust design.

**Figure 10:**
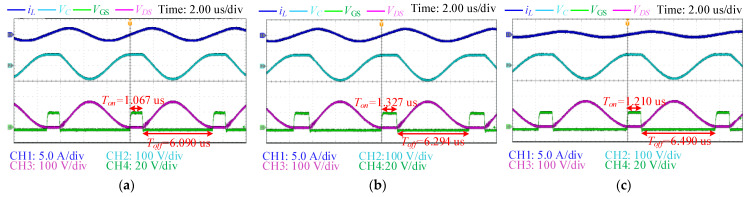
Experimental results to compare the ZVS behaviors under different load resistances: (**a**) *R_L_*= 20 Ω; (**b**) *R_L_* = 43 Ω; (**c**) *R_L_* = 80 Ω.

The critical ZVS conditions of the single-ended resonant converters can be calculated using (18)–(26). Since the switching frequency is up to hundreds of kilohertz, the parameters of the switch are critical for system efficiency, especially when supplying light loads. Since the coil current is reduced, the effect of parasitic capacitance is more notable, and the turn-on time used to achieve soft-switching is significantly increased, as shown in Figure 10. Comparing the waveforms under different load resistances, the turn-on time should be increased to maintain the ZVS condition due to the parasitic capacitance of the switch (about 300 pF for MOSFET). In experiments, the same phenomenon manifests in the increase in turn-on time. 

### 5.3. Efficiency and Output Power

**Figure 11:**
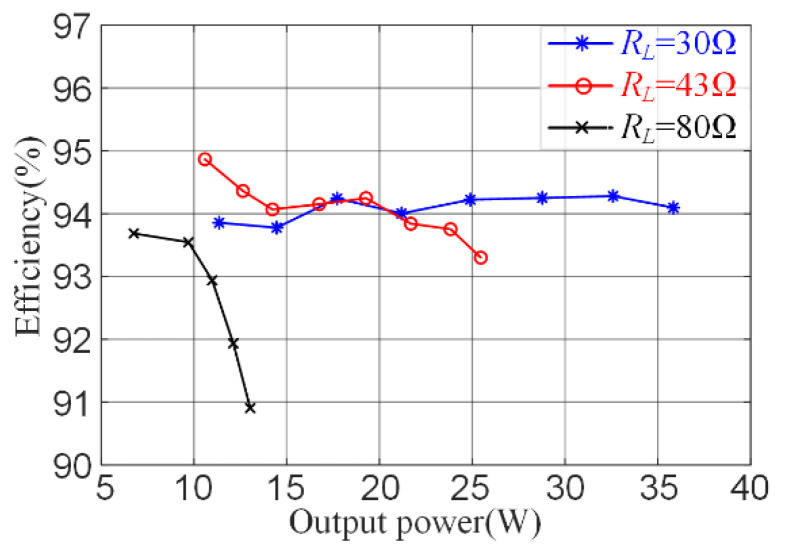
Measured efficiency of the proposed design.

When the operation frequency is up to hundreds of kilohertz, probes can easily introduce additional amplitude and phase errors during the test. Thus, the instantaneous power of the resonant tank will be inaccurate. To estimate the efficiency of the single-ended resonant converter, the average output power of the DC source and the average power delivered to the load resistance were measured. Figure 11 shows the system’s efficiency over the output power range with different load resistances. The driving losses are not considered, and the peak efficiency of the proposed systems is 94.86%. Compared with other topologies, the proposed single-ended WPT system has the advantages of simple circuits, low cost, and easy implementation. It has the potential to be used in small- and medium-power consumer WPT applications.

## 6. Conclusions

This paper presents an analytical model that can be utilized as the theoretical basis for modeling and designing a single-ended resonant converter. Unlike prior work, the proposed single-ended resonant converter replaces the choke inductor of the Class-E inverter with the coil inductance. The time-domain waveform-based circuit model is solved using a numerical approach, which allows the derivation of the design equation for general operating conditions and can hopefully find all feasible designs with the given requirements. Based on the circuit analysis, the detailed parameter design process is given in this paper. The analytical results are found to match the experimental results well, and the system achieved 94.86% peak efficiency at 10.5 W output power at 100 kHz operating frequency. The contributions of this paper are as follows:

1. A single-ended resonant converter to incorporate the coil and the choke inductances of the Class-E inverter, which satisfies both low turn-on switching loss and low turn-off switching loss at the selected power range, is described.
2. The analytical derivation of the operating characteristics and the detailed design procedure are given, including critical ZVS conditions, drain-to-source voltage and input power.

Using the results of this paper, the single-ended resonant converter can be designed for applications with power varying from a few watts to hundreds of watts. Because of its simple structure, the single-ended resonant converter has great advantages for cost-sensitive WPT products, including robot logistics, electric bicycles, and household appliances.

## Figures and Tables

**Table 1:** Value of components used in simulation and experiments.

Parameter	Calculated	Measured
DC supply voltage *V_dc_*	72 V	72.0 V
Input power *P_max_*	25 W	27.3 W
Drain-to-source voltage *V_DSmax_*	252 V	264 V
Switching frequency *f_s_*	100 kHz	100 kHz–136.60 kHz
Primary self-inductance *L_p_*	56.256 uH	57.438 uH/54.17 mΩ
Secondary self-inductance *L_s_*	56.256 uH	56.716 uH/54.05 mΩ
Coupling coefficient *k*	0.35	0.352256
Parallel resonant capacitor *C_p_*	24.339 nF	24.539 nF/100 kHz
Series resonant capacitor *C_s_*	69.513 nF	68.718 nF/100 kHz
Switches	SCT3080AL	600 V/30 A
Diodes	STPS30L60CT	60 V/15 A
Load resistance *R_L_*	43.3012 Ω	43 Ω

## Data Availability

Data available on request from the authors. The data that support the findings of this study are available from the corresponding author, [author initials], upon reasonable request.
